# Apigenin Inhibits Enterovirus-71 Infection by Disrupting Viral RNA Association with *trans*-Acting Factors

**DOI:** 10.1371/journal.pone.0110429

**Published:** 2014-10-16

**Authors:** Wei Zhang, Haishi Qiao, Yuanzi Lv, Jingjing Wang, Xiaoqing Chen, Yayi Hou, Renxiang Tan, Erguang Li

**Affiliations:** 1 Medical School and State Key Laboratory of Pharmaceutical Biotechnology, Nanjing University, Nanjing, Jiangsu, China; 2 College of Life Sciences, Nanjing University, Nanjing, Jiangsu, China; 3 State Key Laboratory of Natural Medicines, China Pharmaceutical University, Nanjing, Jiangsu, China; University of Kansas Medical Center, United States of America

## Abstract

Flavonoids are widely distributed natural products with broad biological activities. Apigenin is a dietary flavonoid that has recently been demonstrated to interact with heterogeneous nuclear ribonucleoproteins (hnRNPs) and interferes with their RNA editing activity. We investigated whether apigenin possessed antiviral activity against enterovirus-71 (EV71) infection since EV71 infection requires of hnRNP proteins. We found that apigenin selectively blocks EV71 infection by disrupting viral RNA association with hnRNP A1 and A2 proteins. The estimated EC_50_ value for apigenin to block EV71 infection was determined at 10.3 µM, while the CC_50_ was estimated at 79.0 µM. The anti-EV71 activity was selective since no activity was detected against several DNA and RNA viruses. Although flavonoids in general share similar structural features, apigenin and kaempferol were among tested compounds with significant activity against EV71 infection. hnRNP proteins function as trans-acting factors regulating EV71 translation. We found that apigenin treatment did not affect EV71-induced nucleocytoplasmic redistribution of hnRNP A1 and A2 proteins. Instead, it prevented EV71 RNA association with hnRNP A1 and A2 proteins. Accordingly, suppression of hnRNP A1 and A2 expression markedly reduced EV71 infection. As a positive sense, single strand RNA virus, EV71 has a type I internal ribosome entry site (IRES) that cooperates with host factors and regulates EV71 translation. The effect of apigenin on EV71 infection was further demonstrated using a bicistronic vector that has the expression of a GFP protein under the control of EV71 5′-UTR. We found that apigenin treatment selectively suppressed the expression of GFP, but not a control gene. In addition to identification of apigenin as an antiviral agent against EV71 infection, this study also exemplifies the significance in antiviral agent discovery by targeting host factors essential for viral replication.

## Introduction

Hand, foot and mouth disease (HFMD) is an infectious disease predominantly caused by enterovirus 71 (EV71) and coxsackievirus A16 (CAV16) of the Picornaviridae family [1]. The disease mainly affects infants and young children of 5 years old or younger with symptoms including skin rashes, watery blisters in the mouth, on the palm, sole, and buttocks. The symptoms are generally self-limited and do not need medications, but those by EV71 infection can have more severe complications, including pulmonary edema, aseptic meningitis or even death. There are over a million reported cases of HFMD with hundreds of deaths in China each year for the last several years [2]. There is an urgent need for the development of therapeutics for HFMD since there is still no vaccine or approved medicine for the prevention or treatment of HFMD [3].

EV71 is a single strand, positive sense RNA virus. The viral genome is composed of approximately 7500 nucleotides that contains a type I internal ribosome entry site (IRES) in the 5′-untranslated region (5′-UTR) essential for translational initiation of the precursor polypeptide upon infection [4], [5]. Host factors that have been identified to modulate EV71 translation include the far-upstream element-binding proteins (FBPs), the heterogeneous nuclear ribonucleoproteins (hnRNPs) [6], [7]. FBP2 functions as a negative regulator and EV71 infection leads to cleavage of FBP2 and therefore enhanced translation of the precursor peptide [8]. Both hnRNP A1 and K proteins have been shown to interact with the stem-loops in the 5′-UTR of EV71 RNA and play an essential role in driving EV71 translation [7], [9], [10]. Combined inhibition of hnRNP A1 and the homologous hnRNP A2 with siRNA suppresses EV71 infection [7]. The anti-EV71 activity of kaempferol was attributed to its ability to alter the composition of IRES associated proteins [11], though no detailed mechanism has been delineated.

hnRNPs are multi-functional proteins regulating several significant steps in the processing of nascent RNA transcripts, including alternative splicing, cytoplasmic RNA trafficking, stabilization, translation of cellular and viral transcripts [6], [12]. They also have been investigated as therapeutic targets for cancer and neurodegenerative diseases [13]–[16]. A recent report by Arango and colleagues showed that apigenin is a selective inhibitor of hnRNP A2 (also known as hnRNP A2/B1, encoded by *hnRNP A2B1* gene). Using a fluorescence probe, combined with chemical biological approaches, they demonstrated that apigenin binds to the C-terminal glycine-rich domain of hnRNP A2 with relatively high affinity. The interaction prevents hnRNPA2 from forming homodimers, and therefore interferes with the function of hnRNP proteins [17]. In light of those important findings, we decided to investigate whether apigenin and structurally-related flavonoids possessed anti-EV71 activity since this may lead to the identification of candidate compounds with a defined target for their antiviral activity.

## Materials and Methods

### Cells and Viruses

African green monkey kidney epithelial cells (Vero) and rhabdomyosarcoma (RD) cells, originally obtained from ATCC (Manassas, VA, USA), were purchased from Cell Bank of Chinese Academy Sciences (Shanghai, China). RD cells were grown in Dulbecco's modified Eagle's medium (DMEM high glucose), supplemented with 10% heat inactivated fetal bovine serum (FBS), *L*-glutamine, nonessential amino acids, sodium pyruvate (Life Technologies). Vero cells were cultured with 10% newborn calf serum (NBCS) instead. Cells were cultured at 37°C in a humidified atmosphere with 5% CO_2_. EV71 Fuyang strain was obtained from Jiangsu CDC. The virus belongs to C4a cluster of the C4 sub-genotype, determined in the lab by sequence analysis of the VP1 region. EV71 was propagated in RD cells. Virus titers were determined in Vero cells by measuring TCID_50_ for EV71 infection using a method by Reed and Muench [18]. Coxsackievirus A16 strain, originally obtained from ATCC, was obtained from China Center for Type Culture Collection (CCTCC) at Wuhan University.

### Antibodies and Reagents

Flavonoids including apigenin, kaempferol, hesperetin, naringenin, and other chemicals used for antiviral studies were purchased from Sigma-Aldrich (St. Luis, MO). Rabbit anti-EV71 VP1 was purchased from Abnova (Taiwan). A mouse antiserum to EV71 was prepared in the lab by immunizing mice with UV-deactivated EV71 and used for immunostaining studies. Antibodies against human hnRNP A1 (BS2255), hnRNP A2/B1 (BS6196), and GAPDH (MB001) were obtained from Bioworld Technology (Minneapolis, MN). Antibody against Lamin B was obtained from Santa Cruz Biotechnology (Dallas, TX). HRP-conjugated secondary antibodies were purchased from Bio-Rad, and Alexa Fluor-conjugated antibodies were from Life Technologies (Carlsbad, CA).

### Cytotoxicity assay

RD cells (2×10^4^) were seeded into 96-well plate and incubated for overnight. Varying concentrations of apigenin at up to 100 µM were added into the medium. The cells were mock treated with DMSO (final concentration of 0.1% or less). Both treated cells and mock treated cells were incubated for 72 hr, followed by incubation with MTT (0.5 mg/mL) for another 4 hr. The ratios of optical densities (ODs) at 570 nm of treated cells over those of untreated cells were used as a measure for percentage of cell viability. Duplicate wells were analyzed for each concentration. CC_50_ was defined as the concentration of apigenin to reduce the viable cells by 50% relative to the untreated control cells and was extrapolated using GraphPad Prism.

### Infection and antiviral assays

For the infection assays, duplicate of RD cells were untreated or pretreated with a compound at concentration as indicated for 2 hr. The cells were then infected with EV71 at an MOI of 0.03 or 0.10 TCID_50_ per cell. We measured the antiviral effect by determining viral VP1 expression directly or by measuring infectious virion production with a secondary infection assay. To assay for infectious virion production, RD cells were pretreated with or without a compound at indicated concentrations for 2 hr and then infected with EV71 at different MOIs as indicated. The cells and cell culture supernatants were collected at 48 hr PI and freeze-thawed 3 times to release infectious virus and assayed on Vero cells to obtain TCID_50_ values.

The EC_50_ value for apigenin antiviral activity was determined by infecting untreated RD cells or those that were pretreated with apigenin at concentrations as indicated 2 hr prior to infection. An MOI of 0.01 TCID_50_ per cell was used with the presence of apigenin throughout the infection. Cell viability was determined using an MTT assay at 72 hr PI. The ratios of OD readings of infected that were treated with or without apigenin over of 40 µM apigenin-treated but uninfected samples were used to calculate the rates of inhibition. Percentage numbers for each point were plotted and used to deduce an EC_50_ value using GraphPad Prism.

### Time of drug-addition study

In time of drug-addition assay, apigenin at 30 µM was added at time points representing time points of viral adsorption, during adsorption/penetration (−2 to 0 hr PI), or post cell entry. An MOI of 0.01 TCID_50_ per cell was used. Apigenin was left in the medium throughout the infection assay. The cytopathic effect was determined by measuring cell viability using MTT at 72 hr PI. An inhibition rate was calculated as a percentage of (OD_treated_–OD_infected_) over (OD_uninfected_–OD_infected_) and was used for the plot.

### Western blotting analysis

Total cell lysates were prepared using a lysis buffer containing 150 mM NaCl, 50 mM Tris-HCl (pH 7.4), 1% NP-40, and a cocktail of protease-inhibitors (Roche). A commercial kit for protein fractionation was purchased from Beyotime Institute of Biotechnology (cat # p0027). The proteins were separated by SDS-PAGE and transferred to PVDF membrane (Millipore). The proteins were detected by immunoblotting analysis using an ECL reagent kit (Millipore). GAPDH was used as an internal control. The images were captured using ChemiScope imaging system (Shanghai, China).

### Immunofluorescence analysis

We followed a reported protocol to study hnRNP A1 and hnRNP A2 distribution [7], [9]. In brief, RD cells were treated with or without 30 µM apigenin for 2 hr and then infected by EV71 at 40 MOI. The cells were fixed with 3% paraformaldehyde at 6 hr PI, followed by permeabilization with 0.2% Triton X-100 in PBS. The cells were stained by incubation with anti-hnRNP A1 or hnRNP A2/B1 antibody and an anti-EV71 antiserum, then with Alexa Fluor 568 conjugated anti-rabbit antibody and Alexa Fluor 488 conjugated anti-mouse antibody. The nuclei were stained with DAPI for 15 min. The images were captured on Olympus BX53 fluorescence microscope (Olympus, Tokyo, Japan) equipped with a QImage CCD camera and the preinstalled software (QImaging, British Columbia, Canada).

### siRNA knockdown

The siRNA or negative control (NC) siRNA was transfected to RD cells using concentrations as indicated with Lipofectamine 2000 (Life Technologies). The cells were harvested for analysis of knockdown effect by western blotting or used for infection study at 60 hr post transfection. siRNAs against hnRNP A1 and hnRNP A2 were synthesized by GenePharma (Shanghai, China) using the following NM_002136 and NM_002137 as references for hnRNP A1 and hnRNP A2, respectively. The sequence information are as following:

hnRNP A1 #1: 5'GGAGGGUUGAGCUUUGAAATT, 5'-UUUCAAAGCUCAACCCUCCTT;

hnRNP A1 #2: 5'-GGUGGAUGCAGCUAUGAAUTT, 5'-AUUCAUAGCUGCAUCCACCTT;

hnRNP A1 #3: 5'-GGCCACAACUGUGAAGUUATT, 5'-UAACUUCACAGUUGUGGCCTT;

hnRNP A2/B1 #1: 5'-GGUGGCUUAAGCUUUGAAATT,

5'-UUUCAAAGCUUAAGCCACCTT;

hnRNP A2/B1 #2: 5'-GGCGGAAUUAAAGAAGAUATT,

5'-UAUCUUCUUUAAUUCCGCCTT;

hnRNP A2/B1 #3: 5'-GGACCAGGAAGUAACUUUATT,

5'-UAAAGUUACUUCCUGGUCCTT.

### Immunoprecipitation assay for associated RNA

We used a method essentially as described to demonstrate apigenin interference with viral RNA association with hnRNP proteins [19]. Briefly, RD cells in 10 cm dishes were treated with apigenin at 30 µM 2 hr prior to EV71 infection or remained untreated. The cells were then infected with EV71 for 6 hr. At the end of infection, the cells were collected by cell scraper. After washing twice with ice-cold PBS, the cells were resuspended in 1 ml PBS and treated with formaldehyde at a final concentration of 0.5% for 5 min. Unreacted formaldehyde was quenched with glycine. The cells were then lysed with a buffer containing 150 mM NaCl, 50 mM Tris-HCl (pH 7.4), 1% NP-40, 0.1% SDS, 1 mM EDTA and protease inhibitors (Roche). Cell lysates (100 µg per sample) were immunoprecipitated with an antibody immobilized on Protein A Sepharose beads. The immunocomplexes were washed with the lysis buffer for 5 times and then heated to 70°C for 30 min to release protein-associated RNAs. The RNAs were collected by isopropanol precipitation after extraction with phenol chloroform to remove proteins.

For RT-PCR studies, the samples were reverse transcribed using a primer that matches the 3′-end of the 5′-UTR region of EV71: 5′-CATGTTTAGCTGTGTTAAGG. Two pairs of primers were used to for PCR amplification (F1: 5′-CCCAGTGAAACTTAGAAGC, R: 5′-CATGTTTAGCTGTGTTAAGG, for SL-II to VI region; F2: 5′-GCACTTCTGTTTCCCCG, R: 5′-CATGTTTAGCTGTGTTAAGG). The reaction was run for 40 cycles at 95°C for 30 s, 58°C for 30 s, and 72°C for 45 s. PCR products were separated by gel electrophoresis and visualized by ethidium bromide staining.

### Plasmid construction and reporter gene assays

We construct a bicistronic vector to demonstrate EV71 5′-UTR as a target of apigenin action using a commercial vector (pIRES2-ZsGreen1, Clonetech) as a template. Briefly, pIRES2-ZsGreen1 was digested with BstXI and then blunted by treatment with Klenow and dNTP mixture by standard molecular biology method. The IRES sequence in the digested vector was then released by further digestion with BamHI and was replaced with EV71 5′-UTR using BamHI and EcoRv. Firefly luciferase gene was then amplified using pGL2 (Promega) as a template and was inserted into the above vector at XhoI/BamHI sites. The resulting plasmid, named pEV-ZsGreen1, hence carries Luc, whose expression is under the control of a CMV promoter, and ZsGreen1 for green fluorescence protein (GFP) following EV71 5′-UTR. The accuracy of the inserted sequences were verified by automated DNA sequencing.

For reporter gene assay, RD cells were plated in a 6-well plate at the density of 2×10^5^/well and allowed to attach overnight. pEV-ZsGreen1 (1 µg/well) was transfected to RD cells using Lipofactamine 2000. We also included pRL (10 ng/well) as an internal control for firefly Luc expression. The cells were treated with 30 µM apigenin or a control compound (naringenin) or 0.1% DMSO since it was used as a solvent 16 hr after transfection. The cells were harvested 24 hr later and GFP expression was determined by FACS analysis on FACS Calibur. The results were analyzed using FlowJo software and the percentage of GFP positive cells was determined. Luciferase expression was determined using Dual-Glo reagent (Promega) and the ratio of firefly and Renila luciferase was calculated and used as a control for comparison of apigenin effect on 5′-UTR activity (% of GFP positive cells over the ratio of firefly luciferase/Renila luciferase).

## Results

### Apigenin possesses antiviral activity against EV71 infection

**Figure 1:**
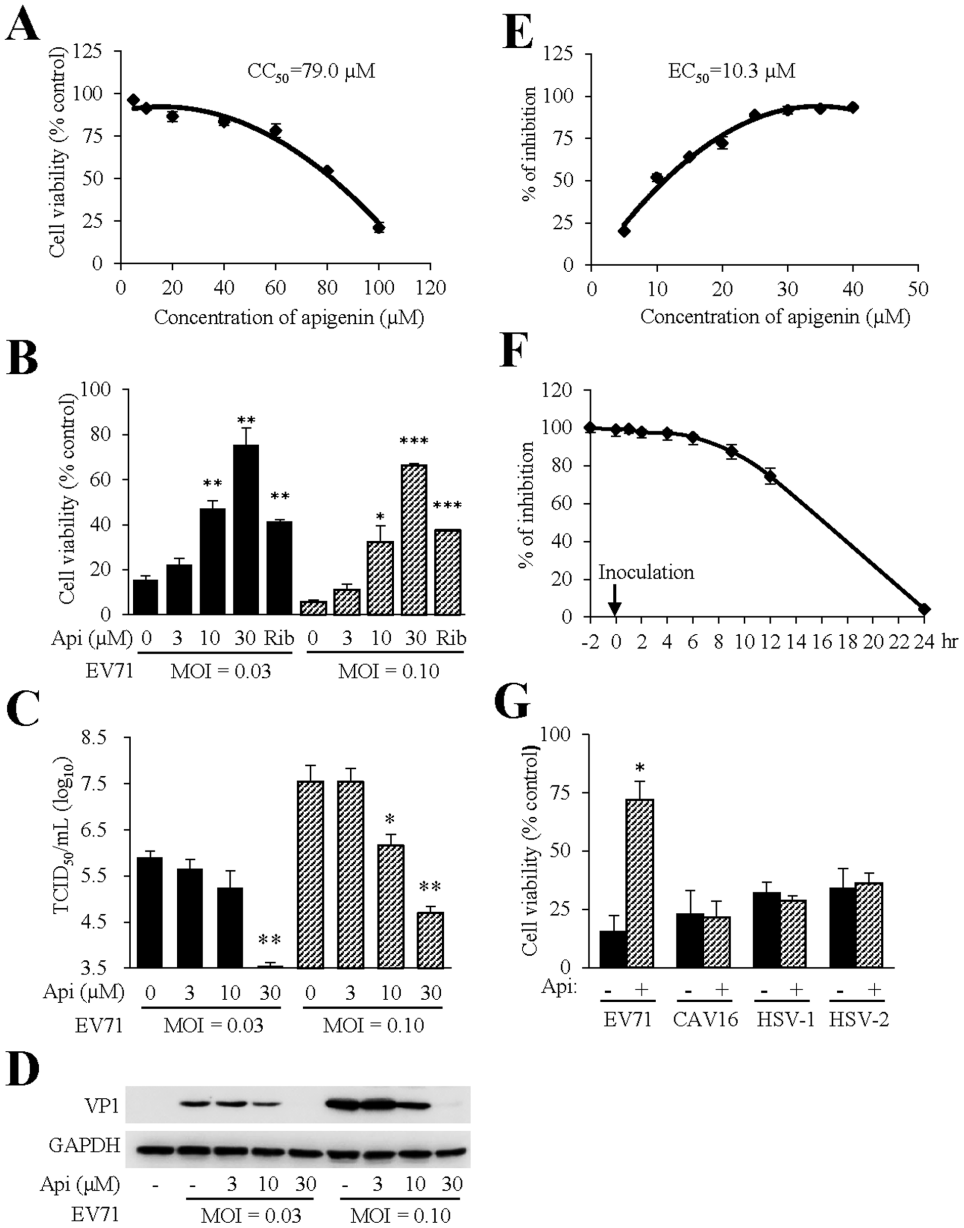
Inhibition of EV71 infection by apigenin. (A) Determination of CC_50_ of apigenin. RD cells in 96 well plates were treated with apigenin at indicated concentrations. Cell viability was assayed at 72 hr post-treatment by measuring MTT reduction. OD readings of duplicate samples were plotted and used to extrapolate CC_50_ values using GraphPad Prism. The experiment was performed two times independently. Data are presented as mean ± standard deviation (SD) of duplicate samples. **(B) Evaluation of apigenin antiviral activity by measuring cytopathic effect.** The permissive RD cells were pretreated 2 hr prior to EV71 infection with apigenin at 3, 10, and 30 µM or mock-treated with DMSO. Ribavirin was included as a positive control. Two MOIs (0.03 and 0.10 TCID_50_ per cell, respectively) were used to infect the cells. The cytopathic effect due to EV71 infection was quantified by measuring cell viability with an MTT assay. The ratios of OD readings of infected samples over uninfected controls were used for this plot. The experiment was performed three times independently. The experiment was performed three times independently and data are presented as mean ± SD of duplicate samples. An unpaired *t* test was performed for statistical analysis. *: *p*≤0.05; **: *p*≤0.01; ***: *p*≤0.001. **(C, D) Validation of apigenin antiviral effect against EV71 infection by titration and immunoblotting for viral VP1 expression.** RD cells were pretreated with apigenin at 3, 10 or 30 µM or remained untreated. The cells were then infected with EV71 at MOI of 0.03 or 0.10 TCID_50_ per cell for 36 hr. Production of infectious virion production (C) or EV71 VP1 protein expression (D) were determined as described in experimental procedures. Titration data are presented as mean ± SD of triplicate samples. The experiment was performed two times independently. An unpaired *t* test was performed for statistical analysis. *: *p*≤0.05; **: *p*≤0.01. GAPDH expression was used as a loading control. **(E) Determination of EC_50_ of apigenin anti-EV71 activity.** RD cells in 96-well plates were treated with apigenin at varying concentrations or remained untreated. The cells were then infected with EV71 at an MOI of 0.01 TCID_50_ per cell for 72 hr. Cell viability was used as a measurement for antiviral effect. Ratios of OD readings of infected over uninfected controls were used for this plot. EC_50_ value was extrapolated using GraphPad Prism. The experiment was performed two times independently. Data are presented as mean ± SD of triplicate samples. **(F) Time of drug-addition antiviral effect of apigenin.** Monolayers of RD cells were pretreated (-2 hr) or treated with 30 µM apigenin at times as indicated. The cells were infected with EV71 at an MOI of 0.01 TCID_50_ per cell for 72 hr. Cell viability was measured using a MTT method. OD reading of triplicate samples at -2 hr of apigenin addition was used as a reference for 100% inhibition, and readings at other times were used to obtain relative inhibition rates at indicated times. The results are representative of two independent experiments. Data are presented as mean ± SD of duplicate samples. **(G) Assay of apigenin antiviral effect against herpes simplex viruses (HSV) and coxsackievirus A16 (CAV16).** Vero cells in triplicate were pretreated with apigenin at 30 µM and then infected with EV71, CAV16, HSV-1 or HSV-2 at an appropriate MOI for each virus for 48 hr. Cell viability was quantitatively measured by MTT assay. The percentages of viable cells were expressed using the ratio of OD readings of infected (solid bar) or infected and treated (hatched bar) samples *vs* uninfected and drug-treated controls. The experiments were performed twice independently. Data are presented as mean ± SD of duplicate samples. An unpaired *t* test was performed for statistical analysis. *: *p*≤0.05.

To investigate whether apigenin had the ability to block EV71 infection, we performed an infection assay using RD cells since the cytopathic effect can be readily assessed microscopically and colorimetrically. To this end, we first determined the nontoxic concentrations that apigenin can be used for the antiviral study. The CC_50_ value of apigenin was determined at approximately 79.0 µM after treatment of RD cells for 3 days (Fig. 1A). We therefore used apigenin at 3, 10, and 30 µM to treat RD cells at 2 hr prior to EV71 infection. In parallel experiment, we also included ribavirin as a positive control. The treated or control-treated cells were then infected with EV71 at two different MOIs (0.03 and 0.10 TCID_50_ per cell, respectively). The infection was monitored under an inverted microscope visually. EV71 at an MOI of 0.10 caused significant cell lysis at 48 hr PI in the untreated controls. For comparison, a significant reduction of cytopathic effect was observed in samples treated with 30 µM apigenin, while apigenin at 3 µM did not visibly affect the morphological changes associated with EV71 infection. The effect was quantitatively measured by assessing cell viability with an MTT assay (Fig. 1B). Compared to that of the infected but untreated controls, cell viability in apigenin-treated (10 and 30 µM) samples were significantly improved, indicating apigenin might possess antiviral activity against EV71 infection.

We next performed a secondary infection assay by measuring infectious virion production to ascertain that the inhibition of cytopathic effect was due to suppression of EV71 infection. As shown in Fig. 1C, apigenin at 30 µM significantly reduced infectious virion production at two different MOIs (by approximately 2.4 and 2.9 logs, respectively), while the effect at 10 µM was less effective, but significant, in the higher MOI group. The inhibition on infectious EV71 production was substantiated with the detection of reduced VP1 protein expression in apigenin-treated samples (Fig. 1D). VP1 expression was markedly reduced in one set of samples treated with 10 µM apigenin, while the expression of VP1 was completely blocked in samples treated with 30 µM apigenin. The results clearly demonstrated that apigenin had antiviral activity against EV71 infection.

The antiviral effect of apigenin against EV71 infection was quantitatively measured by the determination of a 50% percent effective concentration (EC_50_). The EC_50_ was estimated at 10.3 µM (Fig. 1E), significantly lower than the reported data for ribavirin (approximately 266 µM) in a previous study [20]. In addition, a time of drug-addition study was performed. As shown in Fig. 1F, pretreatment of RD cells or within the first 6 hr of inoculation with apigenin at 30 µM resulted in suppression of EV71 infection to comparable levels. We interpreted this as that apigenin inhibited EV71 infection by targeting a post cell entry event(s).

As a common dietary flavonoid, apigenin is widely distributed in fruits and vegetables. We therefore extended this finding by investigating whether apigenin possessed antiviral activity against several viruses of human diseases, including herpes simplex virus-1 and -2 (HSV-1, HSV-2), coxsackievirus A16, a pathogen of HFMD with mild symptoms. At up to 30 µM concentrations, apigenin showed no antiviral effect against those viruses (Fig. 1G), indicating the antiviral effect of apigenin against EV71 infection was specific.

### Apigenin disrupts EV71 RNA association with hnRNP proteins

**Figure 2:**
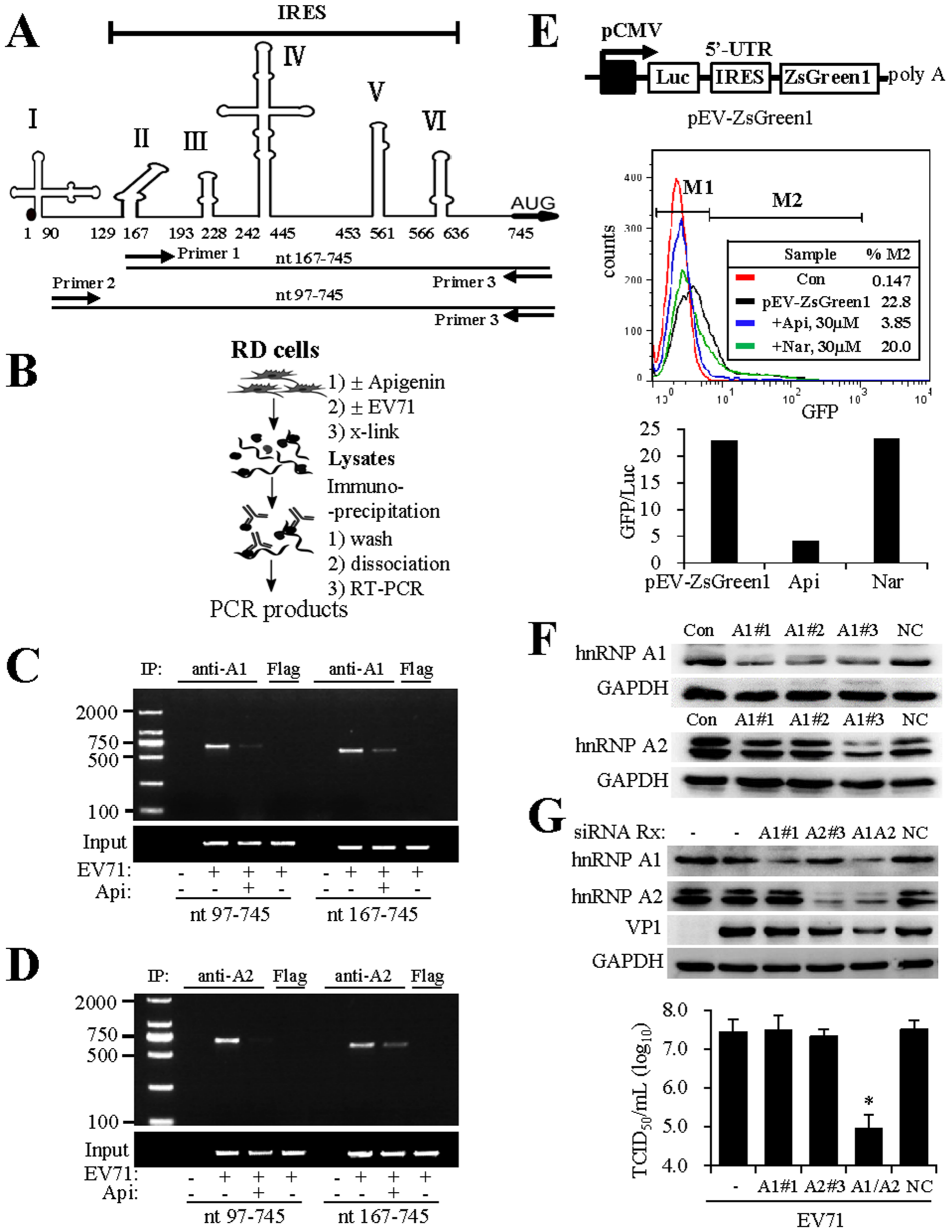
Apigenin disrupts EV71 RNA association with hnRNP A1 and A2 proteins. (A) Diagram of the 5′-UTR region of EV71 genome. The 5′-UTR region of EV71 strain used in this study was sequenced and used for the prediction of secondary structure using MFOLD program and presented using ChemBioDraw. The numbers indicate relative position of nucleotides. Primers 1 to 3 indicate positions of corresponding oligos used for genome detection by RT-PCR. **(B) Schematic drawing of experimental procedures of cross-link followed by immunoprecipitation and RT-PCR amplification for hnRNP-associated viral RNA.** **Detection of EV71 RNA association with hnRNP A1 (C) and hnRNP A2 (D) proteins.** RD cells were pretreated with 30 µM apigenin or untreated. The cells were then infected with EV71 at an MOI = 40 for 6 hr. hnRNP-associated viral RNA was detected using RIP followed by RT-PCR. Anti-FLAG antibody was used as a control for immunoprecipitation. In those experiments, portions of the cell lysates were used to determine total RNA input by RT-PCR. Two sets of oligos (nt 97–745 and 167–745, respectively) were used for the detection of hnRNP-associated EV71 RNA. Data are representative of three independent experiments. **(E) Suppression of apigenin on IRES activity.** A bicistronic reporter gene system (diagram) was constructed for testing EV71 IRES activity. The plasmid, named pEV-ZsGreen1, was transfected to RD cells, using pRL as an internal control for firefly luciferase expression (refer to materials and methods). The expression of ZsGreen GFP was quantitatively measured by FACS analysis (middle panel). The expression of firefly luciferase and Renila luciferase was determined using Dual-Glo reagent. The percentages of GFP positive cells in apigenin-treated or control samples were plotted against luciferase activity (lower panel, arbitrary number using untreated pEV-ZsGreen1 as 1). The experiment was performed two times independently. **(F) Screening of siRNAs that suppress hnRNP A1 or hnRNP A2 expression.** RD cells in 24-well plates were transfected with a scrambled siRNA (NC, at 50 pmol/well) or an siRNA (50 pmol/well) targeting hnRNP A1 (named as A1#1 to 3) or hnRNP A2 (named as A2#1 to 3) expression. The sequences for those oligos are given in experimental procedures. The cells were harvested at 60 hr post transfection and protein expression was detected by immunoblotting. GAPDH expression was used as a control. A1#1 and A2#3 were selected and used for knockdown hnRNP A1 and hnRNP A2 studies. Results are representatives of three independent experiments. **(G) Suppression of hnRNP A1 and A2 expression by siRNA inhibits EV71 infection.** RD cells in a 24-well plate were transfected with a scrambled control siRNA (NC, 70 pmol/well), siRNA for suppression of hnRNP A1 (A1#1, 35 pmol A1#1 plus 35 pmol NC siRNA), hnRNP A2 (A2#3, 35 pmol A2#3 plus 35 pmol NC siRNA), or 35 pmol each of A1#1 and A2#3 (A1+A2) for combined suppression of hnRNP A1 and A2 expression. The cells were fed with fresh medium at 60 hr post transfection and then infected with EV71 (MOI = 0.10) for another 36 hr. Protein expression was detected by immunoblotting. In parallel experiments, the samples were harvested and infectious virion production was determined by titration. Results are representatives of two independent experiments. Data are presented as mean ± SD of triplicate samples. An unpaired *t* test was performed for statistical analysis. *: *p*≤0.05.

A recent study demonstrated that apigenin directly binds to hnRNP A2 protein and perturbs with its function. In consideration of the importance of hnRNP proteins in translational initiation of EV71 infection, we next investigated whether apigenin targeted hnRNP proteins for its anti-EV71 activity. To this end, we performed a cross-link followed by immunoprecipitation and RT-PCR assay [19]. Apigenin-treated RD cells or untreated controls were infected with EV71 at an MOI = 40, a relative high concentration used for the investigation of the early events of EV71 infection [7], [9]. After cross-linking, the cells were then lysed and host protein-associated viral RNA was pulled down by immunoprecipitation with antibodies to human hnRNP A1, A2 or a control antibody, and viral RNA in the immunocomplexes was detected by RT-PCR studies using two pairs of primers that anneal to the 5′-UTR region of EV71 genome (Fig. 2A, Fig. 2B). As shown in Fig. 2C and 2D, EV71 RNA was associated with hnRNP A1 and A2. Treatment with apigenin resulted in significant reduction of EV71 RNA in the immunocomplexes, even though the treatment did not significantly affect the levels of EV71 total RNA in those samples (input control). Those results suggested that apigenin potentially targets viral RNA association with hnRNP proteins, essential factors for EV71 infection.

The essential role of 5′-UTR in apigenin anti-EV71 infection was demonstrated using a reporter gene assay. We first constructed a bicistronic vector that carries both firefly luciferase (Luc) and ZsGreen1 green fluorescence protein (Fig. 2E, diagram). The expression of Luc was under the control of a CMV promoter, while GFP expression was under the control of EV71 IRES instead of ECMV IRES in the original vector from Clonetech. The expression of GFP in apigenin-treated or control-treated samples was determined by FACS analysis. As shown in Fig. 2E, treatment with apigenin, but not a control flavonoid naringenin, significantly suppressed GFP expression. In contrast, the treatment showed little effect on Luc whose expression was under the control of a CMV promoter. This result supported the conclusion that apigenin targeted the 5′-UTR for its antiviral effect.

The importance of hnRNPs in EV71 infection was also demonstrated with the use of siRNA. As shown in Fig. 2F and 2G, knockdown of hnRNP A1 or A2 individually did not significantly affect EV71 infection, possibly due to the fact that those two proteins are products of gene duplication and their expression is generally redundant in most cells and tissues [7], [21]. Combined knockdown of both hnRNP A1 and A2 by co-transfection significantly inhibited infectious virion production. Those results therefore strengthen the conclusion that hnRNP proteins play a critical role in apigenin action against EV71 infection.

### Apigenin treatment does not affect hnRNP redistribution caused by EV71 infection

**Figure 3:**
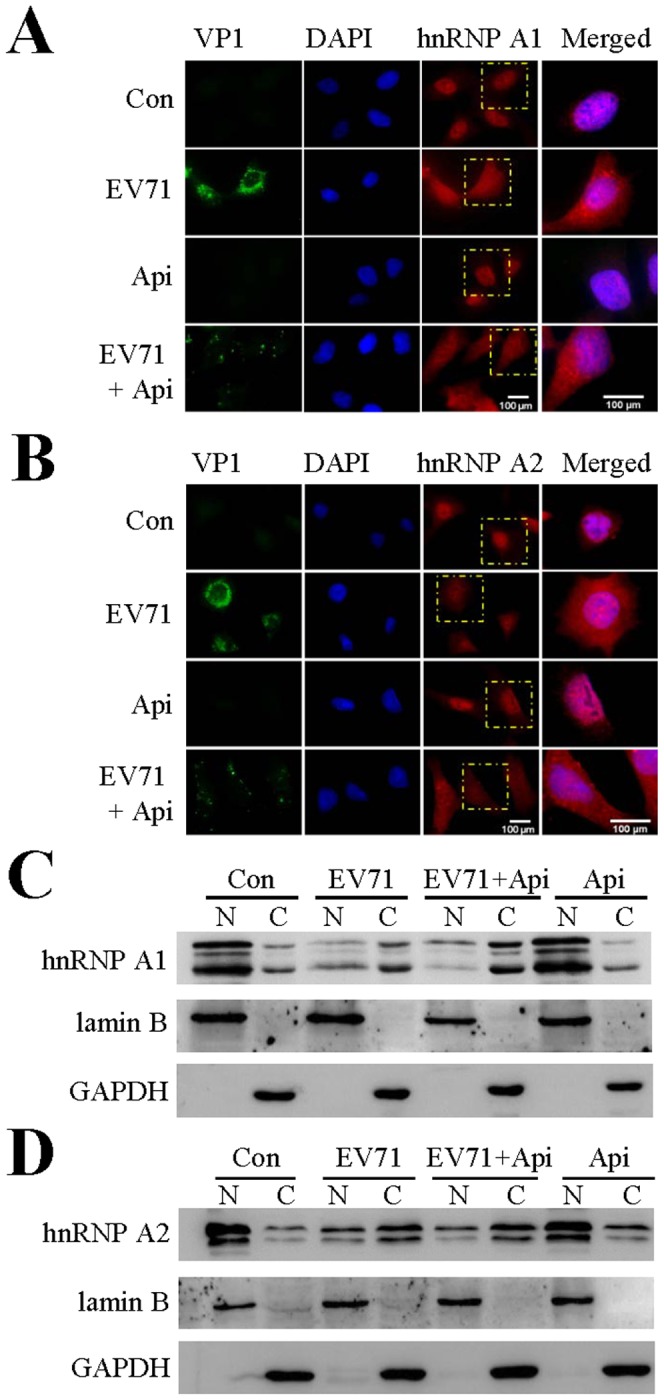
Apigenin treatment does not affect hnRNP A1 or hnRNP A2 nucleocytoplasmic redistribution induced by EV71 infection. (A, B) Immunostaining study to determine hnRNP A1 and hnRNP A2 redistribution. RD cells on coverslips were untreated or treated with 30 µM apigenin 2 hr prior to EV71 infection. The cells were infected with EV71 for 6 hr at an MOI of 40. The cells were then fixed and stained with rabbit anti-hnRNP A1 (A, red) or anti-hnRNP A2 (B, red), followed by Alexa Fluor 568-conjugated anti-rabbit antibody. EV71 VP1 protein expression was stained with a mouse anti-EV71 antibody (green) and the corresponding secondary antibody. The nuclei were visualized by staining with DAPI (blue). The merged images represent the areas within the yellow squares and highlight the relative location of hnRNP to the nuclei. (A) EV71 infection causes hnRNP A1 redistribution, while apigenin treatment does not affect hnRNP A1 redistribution. (B) EV71 infection causes hnRNP A2 redistribution and apigenin treatment does not affect hnRNP A2 redistribution. Images were collected with 400x magnifications and were processed using Image J. **(C, D) hnRNP A1 and A2 redistribution by fractionation studies.** Apigenin treated or control RD cells were uninfected or infected with EV71 at an MOI of 40 for 6 hr. The cells were harvested and fractionated for detection of cytosolic and nuclear proteins by immunoblotting. (C) redistribution of hnRNP A1 protein and (D) redistribution of hnRNP A2 protein. Lamin B and GAPDH were used as loading controls for nuclear and cytoplasmic proteins, respectively. Results are representatives of two independent experiments.

hnRNP proteins are important factors in RNA editing, stability and transportation [22]. We also investigated whether apigenin treatment affected hnRNP A1 and A2 redistribution by immunostaining studies since nucleocytoplasmic redistribution has been reported during EV71 infection [7]. hnRNP A1 and A2 proteins were detected mainly in the nuclei of uninfected or apigenin-treated cells (Fig. 3A, 3B). EV71 infection promoted the redistribution of those proteins to the cytosol. Although apigenin treatment disrupted viral RNA association with hnRNP A1 and A2 (refer to Fig. 2C, 2D), the treatment showed no effect on hnRNP redistribution caused by EV71 infection (Fig. 3A, 3B), a conclusion supported by results from protein fractionation studies (Fig. 3C, 3D). Those results demonstrated that apigenin inhibits EV71 infection by blocking viral RNA association with hnRNP A1 and A2, essential factors for initiation of EV71 polypeptide translation.

### Antiviral activity evaluation of flavonoids

**Figure 4:**
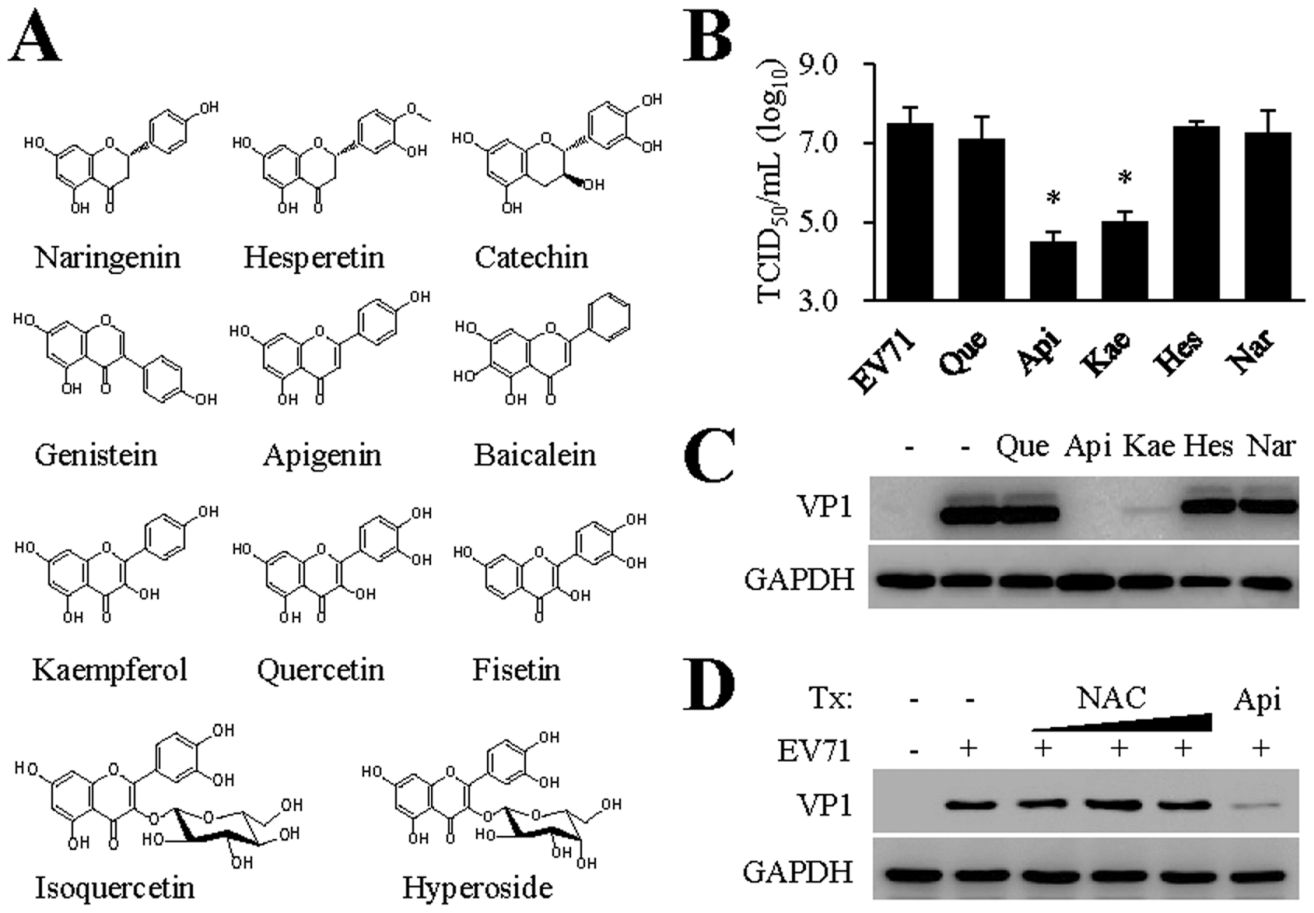
Antiviral activity evaluation of flavonoids against EV71 infection. (A) Names and chemical structures of flavonoids tested in this study. The compounds were evaluated for their antiviral activity against EV71 infection. Apigenin and kaempferol were the only compounds that showed antiviral activity at 30 µM concentration. **(B, C) Confirmation of kaempferol antiviral activity by titration for infectious virion production (B) and viral VP1 protein expression (C).** RD cells were untreated or pretreated with kaempferol along with apigenin, naringenin and hesperetin at 30 µM for 2 hr and then infected with EV71 (MOI = 0.10) for 36 hr. Infectious virion production was titrated using a secondary infection assay (B) and VP1 expression was detected by immunoblotting (C). Que: quercetin; Api: apigenin; Kae: kaempferol; Hes: hesperetin; Nar: naringenin. GAPDH expression was used as a loading control. Both studies were performed twice independently. Data are presented as mean ± SD of triplicate samples. An unpaired *t* test was performed for statistical analysis. *: *p*≤0.05. **(D) Anti-oxidative agent** ***N*** **-acetyl cysteine (NAC) treatment does not affect EV71 infection.** RD cells were untreated or pretreated with NAC at varying concentrations (1, 3, and 10 mM) or apigenin at 30 µM for 2 hr. The cells were then infected with EV71 (MOI = 0.10 TCID_50_ per cell) for 36 hr. EV71 VP1 protein expression were determined by immunoblotting analysis. The experiment was performed twice independently. GAPDH was used as a loading control.

Finally, we investigated whether flavonoids with similar structural features possessed antiviral activity against EV71 infection. Monolayers of RD cells were treated with a panel of flavonoids (Fig. 4A) at 30 µM prior to EV71 infection. Interestingly, apigenin along with kaempferol, a compound recently reported to inhibit EV71 infection by altering trans-acting factor binding [11], were the only compounds showed activity against EV71 infection. The antiviral effect of kaempferol was nevertheless validated by titration of infectious virion production (Fig. 4B) and by viral protein expression (Fig. 4C). In addition to reduction of virus titers, treatment with kaempferol, but not naringenin, quercetin, or hesperetin, resulted in significant reduction of VP1 protein expression (Fig. 4C). Although the anti-oxidant activity of polyphenols has commonly be attributed to their antiviral property, the fact that apigenin and kaempferol were among the only compounds with antiviral activity against EV71 infection argues strongly against anti-oxidative property for apigenin antiviral activity. Indeed, *N*-acetyl cysteine, a commonly used anti-oxidative agent, showed no effect against EV71 infection (Fig. 4D).

## Discussion

We showed here that apigenin, a dietary flavonoid, possesses antiviral activity selective to EV71 infection. Apigenin is a common polyphenolic compound with antioxidant property. Our data seem to indicate otherwise that the compound utilizes a distinct mechanism for its antiviral activity against EV71 infection. We showed that apigenin treatment disrupted viral RNA association with host trans-acting factors, representing a previously undescribed mechanism for apigenin antiviral activity against EV71 infection.

The genome of EV71 has a well characterized IRES for the regulation of translational initiation upon EV71 infection. Host factors including FBPs and hnRNPs have been demonstrated as essential factors involved in EV71 translation through their interaction with the IRES [6], [7]. hnRNP A1 interacts with the stem-loop structural elements within the IRES with high affinity and trans activates EV71 RNA translation [10]. The virus removes negative regulator effect of FBP2 through proteolytic cleavage of FBP2 protein, resulting in enhanced translation of the precursor peptide [8]. Through high throughput screening combined with biochemical approaches, Arango and colleagues identified hnRNP A2 as a putative target of apigenin function [17]. The human hnRNP A1 and A2 proteins share significant similarity since these are products of two homologous genes originated by duplication [21]. We found that EV71 RNA associates with both hnRNP A1 and A2. Inclusion of apigenin during EV71 infection significantly reduced hnRNP A1 and A2 associated viral RNA, even though no obvious reduction of EV71 total RNA in the treated samples, underlying the importance of hnRNP proteins in EV71 infection. This study also suggested that small molecules that target viral RNA association with trans-acting factors may serve as a new strategy for antiviral agents [3]. In addition to our study reported here, kaempferol is another example that has been shown to inhibit EV71 infection by changing the composition of IRES associated proteins [11].

It is interesting to note that the antiviral effect of apigenin was selective against EV71, but not CAV16. Both EV71 and CAV16 are classified into the same cluster based on sequence information [23], [24]. It is known that the 5′-UTR region sequences of enteroviruses are highly conserved (summarized in Siafakas et al. 2002). Although the 5′-UTR region of EV71 and CAV16 have about 85% identity [23], a sequence alignment of this region of CAV16 and EV71 shows more significant difference in the SL-II and SL-VI loops, regions that have more prominent role in EV71 translation and infection [10]. It has been observed that subtle difference in SL-II region can have significant difference of virulence of EV71 in mice [25]. The sequence differences of the 5′-UTR regions of EV71 and CAV16 might be the underlying mechanisms for selective antiviral effect of apigenin against EV71 infection.

HFMD is a contagious disease that affects millions of children of kindergarten ages. Recent outbreaks with high incidence of cardiopulmonary and neurologic complications by EV71 infection have become more prevalent in the Asia Pacific region. Despite profound effort by government agencies and public awareness, the disease still causes hundreds of deaths of children each year due to the lack of remedies. Flavonoids are widely distributed in plants and have broad activities with anti-cancer, anti-oxidative, anti-inflammatory, and antiviral effects [26]–[28]. Although several flavonoids have also been reported with activity against enterovirus and hepatovirus, including EV71, poliovirus, some group A and B coxsackieviruses, echovirus 6, and hepatitis A virus infections [29]–[31], the effective concentrations tend to be significantly higher than those by apigenin and kaempferol, possibly due to lack of specific targets of action. The fact that apigenin directly interacts with hnRNP A2 would suggest a strict structural requirement for apigenin action. Indeed, compounds that share significant structure similarity to apigenin showed no activity against EV71 infection. This study therefore uncovers a previously undescribed mechanism of flavonoid antiviral activity. This study may also serve as a proof-of-concept for the discovery of antiviral agents by targeting host factors essential for virus replication and assembly.
